# Multiplex recombinase polymerase amplification for high-risk and low-risk type HPV detection, as potential local use in single tube

**DOI:** 10.1038/s41598-023-28038-9

**Published:** 2023-01-16

**Authors:** Rungdawan Wongsamart, Parvapan Bhattarakasol, Arkom Chaiwongkot, Doonyapong Wongsawaeng, Pilailuk Akkapaiboon Okada, Tanapat Palaga, Asada Leelahavanichkul, Weerapan Khovidhunkit, Deborah Dean, Naraporn Somboonna

**Affiliations:** 1 Department of Microbiology, Faculty of Science, Chulalongkorn University, Phyathai Road, Pathumwan, Bangkok, 10330 Thailand; 2 Microbiome Research Unit for Probiotics in Food and Cosmetics, Chulalongkorn University, Bangkok, 10330 Thailand; 3 Department of Microbiology, Faculty of Medicine, Chulalongkorn University, Bangkok, 10330 Thailand; 4 Center of Excellence in Applied Medical Virology, Department of Microbiology, Faculty of Medicine, Chulalongkorn University, Bangkok, 10330 Thailand; 5 Department of Nuclear Engineering, Faculty of Engineering, Chulalongkorn University, Bangkok, 10330 Thailand; 6 National Institute of Health of Thailand, Department of Medical Sciences, Ministry of Public Health, Nonthaburi, 11000 Thailand; 7 Department of Medicine, Faculty of Medicine, Chulalongkorn University, Bangkok, 10330 Thailand; 8 Center for Immunobiology and Vaccine Development, UCSF Benioff Children’s Hospital Oakland Research Institute, Oakland, CA 94609 USA; 9 Department of Medicine, University of California, San Francisco, CA 94143 USA; 10 UC Berkeley/UCSF Graduate Program in Bioengineering, University of California, Berkeley, CA 94720 USA; 11 Omics Sciences and Bioinformatics Center, Faculty of Science, Chulalongkorn University, Bangkok, 10330 Thailand

**Keywords:** Biological techniques, Biotechnology, Microbiology, Molecular biology, Health care

## Abstract

High rates of new cervical cancer cases and deaths occur in low- and middle-income countries yearly, and one reason was found related to limitation of regular cervical cancer screening in local and low-resource settings. HPV has over 150 types, yet certain 14–20 high-risk and 13–14 low-risk types are common, and, thus, most conventional HPV nucleic acid assays, for examples, Cobas 4800 HPV test (Roche Diagnostics, New Jersey, USA) and REBA HPV-ID (Molecules and Diagnostics, Wonju, Republic of Korea) were developed to cover these types. We thereby utilized bioinformatics combined with recent isothermal amplification technique at 35–42 °C to firstly describe multiplex recombinase polymerase amplification assay that is specific to these common 20 high-risk and 14 low-risk types, and also L1 and E6/E7 genes that target different stages of cervical cancer development. Multiplex primer concentrations and reaction incubation conditions were optimized to allow simultaneous two gene detections at limit of detection of 1000 copies (equivalent to 2.01 fg) for L1 and 100 copies (0.0125 fg) for E6/E7, respectively. The assay was validated against urogenital and other pathogens, normal flora, and human control. In 130 real clinical sample tests, the assay demonstrated 100% specificity, 78% diagnostic accuracy, and 75% sensitivity compared with REBA HPV-ID test, and is much more rapid (15–40 min), less expensive (~ 3–4 USD/reaction) and does not require instrumentation (35–42 °C reaction condition so hand holding or tropical temperature is possible). Hence, the developed novel assay provides alternative screening tool for potential local screening. Furthermore, as this assay uses safe chemical reagents, it is safe for users.

## Introduction

Cervical cancer is a major public health problem in developed and developing countries. According to the World Health Organization (WHO), an estimated 604,000 new cases and 342,000 deaths of cervical cancer occurred worldwide in 2020, and 90% of these cases were in low- and middle-income countries^1,2^. The reasons were found primarily related to a smaller rate of access to cervical cancer vaccination as a preventive venue and to regular cervical cancer screening programs (and, thus, screening-positive women could be treated adequately before cancer development) compared with high-income countries^2^. In Thailand and other Southeast Asia countries, cervical cancer represents the most common cancer among women. The Institute of Oncology/International Agency for Research on Cancer reported that ~ 9158 women were diagnosed with cervical cancer and 4705 died every year, and estimated approximately 3.4% of the Thai women population carried human papillomavirus (HPV) high-risk types 16 or 18^3^. In addition, the cost of radiation therapy for cervical cancer and treatment of side effects, such as gastrointestinal or genitourinary side effects, is as high as ~ 63,103 USD per patient. The 2018 economic burden for merely radiation therapy in Thailand was ~ 131 million USD, and for the diagnosis of these advanced cervical cancer patients was ~ 129 million USD^4^. Subsequently, an effective, simple, easy-access, and low-price screening method for early prevention is important to minimize the number of cases and the economic burden of advanced cervical cancer patients.

More than 95% of cervical cancer are caused by HPV. HPV is also associated with more than 60–90% of anal cancer, vaginal cancer, oropharyngeal cancer, and penile cancer^5–7^. HPV has over 150 types and is classified into high-risk (HR) or low-risk (LR) for cervical cancer development. Common HPV HR types include 16, 18, 31, 33, 34, 35, 39, 45, 51, 52, 58, 59, 68, and 82, given types 16 and 18 generally account for 70% of cervical cancer. Common HPV LR types include 6, 11, 32, 40, 42, 43, 44, 54, 61, 70, 72, 81, 84, and 87^2,5,8,9^.

Traditionally, a cytological examination by Papanicolaou test (Pap smear) is simple and established. However, the procedure is inconvenient, cannot be performed in local resource- and clinician-limited settings, and its sensitivity (i.e., the limit of detection) and specificity to detect precancerous or cancerous changes in cells are relatively low compared with nucleic acid amplification test^10,11^. For instance, Spence et al.^12^ reported a false negative rate of Pap smear to be 35.5% on average with the sensitivity ranging from 30 to 87%^11^. Furthermore, the Pap smear may require another HPV-specific nucleic acid test, such as Cobas 4800 HPV test (Roche Diagnostics, New Jersey, USA) and REBA HPV-ID (Molecules and Diagnostics, Wonju, Republic of Korea), as accompaniment and identification of HR types. Therefore, the HPV-specific tests were suggested as an alternative screening tool and some even reported that these tests could allow screening intervals to be extended to ≥ 5 years^11,13–15^. The Cobas 4800 HPV test (price ~ 8.5 USD, time ~ 5 h per reaction) utilizes amplification of target DNA by polymerase chain reaction (PCR) and nucleic acid hybridization for detection of HPV HR types 16, 18, and a pool of 31, 33, 35, 39, 45, 51, 52, 56, 58, 59, 66, and 68^15^. REBA HPV-ID (price ~ 25.2 USD, time ~ 6 h per reaction) is a PCR-based reverse blot hybridization assay for 19 HPV HR probes (types 16, 18, 26, 31, 33, 34, 35, 39, 45, 51, 52, 53, 56, 58, 59, 66, 68, 69, and 73) and 13 LR probes (types 6, 11, 32, 40, 42, 43, 44, 54, 70, 72, 81, 84, and 87) for HR and LR genotyping^16^. Although the Cobas and REBA assays are available commercially and used widely in in vitro clinical laboratories, they require an expensive thermal cycler for PCR amplification of HPV DNA and a special instrument for product-probe detection^15,16^.

Recently, an isothermal amplification assay like recombinase polymerase amplification is emerged to solve this problem. The recombinase polymerase amplification utilizes 3 enzymes, a recombinase, a single-stranded DNA-binding protein, and strand-displacing polymerase, to allow isothermal amplification of specific primer-binding target(s) at 37–42 °C. This allows the reaction to be simply performed by a heat block or water bath, or even holding a tube in hands, supporting low cost, rapid and point-of-care molecular tests^17^. For HPV detection, Ma et al.^18^ developed RPA for HPV HR types 16 and 18, and Gong et al.^19^ developed RPA for 13 HPV HR types based on the L1 gene. We, thereby, further developed a multiplex recombinase polymerase amplification (mRPA) assay with attempt to cover broad HPV HR and LR types, as well as normal and precancerous stages of HPV developmental cycle in humans, as potential local use in a single tube for broad and precancerous screenings of cervical cancer. We determined the limit of detection, validated proper specificity on positive controls comprising some HR and LR types and negative controls comprising urogenital bacteria, other viruses, human and mouse, and evaluated the assay efficiency (e.g., sensitivity, specificity, and accuracy) on real 130 clinical cervical swab samples compared with the Cobas and REBA results. In HPV developmental cycle, a late gene L1 encodes for a major viral capsid protein, while early genes E6 and E7 encode for oncoproteins and their detection suggests an increased risk of cancer development (e.g., the higher E6/E7 than L1 mRNA expression was found in cancer patients). E6 degrades a human growth suppressor protein p53 and E7 degrades a human retinoblastoma tumor suppressor protein pRb, and, thus, the E6 and E7 induce cancer development by evasion of human growth suppressors, resisting cell death, sustaining proliferative signaling, enabling replicative immortality, angiogenesis induction, and activation of invasion and metastasis^20^. Consequently, this study designed multiplex degenerate primers based on HPV L1 and E6/E7 genes, covering up to 20 HR and 14 LR types, and the E6/E7 amplification product could suggest an increased risk of cancer.

## Materials and methods

### mRPA primers and design

All GenBank available HPV type L1 (and E6/E7) sequences were aligned using ClustalW (www.megasoftware.net/), and RPA degenerate primers were designed using Primer-BLAST^21^ or PrimedRPA^22^ and manual design (Table 1). Of each gene, a cocktail of more than one degenerate primer sequences were used to allow primer coverage of up to 20 HPV HR types (16, 18, 26, 31, 33, 34, 35, 39, 45, 51, 52, 53, 56, 58, 59, 66, 68, 69, 73, and 82) and 14 LR types (6, 11, 32, 40, 42, 43, 44, 54, 61, 70, 72, 81, 84 and 87). Each primer specificity was verified by BLASTN. The primers were synthesized by Macrogen Inc., Korea.

**Table 1:** RPA primers and their HPV type coverage.

Primers	Sequences (5′ → 3′)	HPV type coverage	
		High-risk (HR)	Low-risk (LR)
RPA_L1_F	GCCCAGGGMCAYAATAATGGTATWTGCTGG	16, 18, 26, 31, 33, 34, 35, 39, 45, 51, 52, 53, 56, 58, 59, 66, 68, 69, 73, 82	6, 11, 32, 40, 42, 43, 44, 54, 61, 70, 81, 84, 87
RPA_L1_R1	CAYAATTGAAAAATAAATTGYAAATCAWACTCCTC	16, 18, 26, 31, 35, 45, 51, 52, 56, 82	6, 11, 40, 61, 87
RPA_L1_R2	CACARYTGAAATATAAAYTGYAAATCATATTCCTC	16, 18, 31, 35, 39, 45, 51, 52, 53, 58, 59, 66, 68, 82	6, 11, 32, 42, 43, 44, 54, 70, 72, 81, 84
RPA_E6/E7_F	GAGGTATATGAYTTTGCTTTTCSWGATTTA	16, 18, 33, 34, 35, 39, 45, 51, 52, 56, 68, 69, 73, 82	6, 11, 40, 42, 43, 44, 54, 61, 70, 72
RPA_E6/E7_R1	AATWATAATGTCTATACTCMCTWATTTTAG	16, 33, 34, 35, 45, 51, 52, 53, 58	6, 11, 40, 42, 43, 44
RPA_E6/E7_R2	TTCTATACTATCTAAATTCTCTTACTCTTG	31, 33, 35, 39, 45, 58, 59, 68, 69, 73, 82	6, 11, 54, 61, 70, 72
RPA_E6/E7_R3	ATTTATAAYGYCTAAATTCACTTATTTTAGA	16, 31, 35, 52, 58	
RPA_E6/E7_R4	ATAMTKTCTGAATTCTCTAATTCTAGAATAAAA	18, 45	

### Reference and clinical strains

Microorganisms used in this study included 12 negative samples comprising bacteria that are sexually transmitted disease pathogens (*Chlamydia trachomatis, Neisseria gonorrhoeae*, and *Staphylococcus saprophyticus*), urogenital normal flora (*Staphylococcus aureus* and *Escherichia coli*), skin normal flora (*Staphylococcus epidermidis*), viruses, and mammals (Table 2). These references were provided by the Buddhachinaraj Phitsanulok Hospital; Department of Medical Sciences, Ministry of Public Health; Department of Microbiology, Faculty of Science, Chulalongkorn University; and Faculty of Medicine, Chulalongkorn University^23–26^.

**Table 2:** List of bacteria, virus, and mammal strains used as negative samples in specificity test.

Strains	Sources and/or references
Bacteria	
*Chlamydia trachomatis* DNA	Buddhachinaraj Phitsanulok Hospital^23^
*Neisseria gonorrhoeae* DNA	Buddhachinaraj Phitsanulok Hospital^23^
*Staphylococcus saprophyticus* ATCC15305	Department of Medical Sciences, Ministry of Public Health^24^
*Staphylococcus aureus* ATCC25928	Department of Microbiology, Faculty of Science, Chulalongkorn University^24^
*Staphylococcus epidermidis*	Department of Microbiology, Faculty of Science, Chulalongkorn University
*Escherichia coli* ATCC25922	Department of Microbiology, Faculty of Science, Chulalongkorn University^26^
Virus	
Hepatitis B DNA	Department of Microbiology, Faculty of Science, Chulalongkorn University
SARS-CoV-2 (oligo synthetic DNA)	Department of Medical Sciences, Ministry of Public Health
Influenza A (oligo synthetic DNA)	Department of Medical Sciences, Ministry of Public Health
Influenza B (oligo synthetic DNA)	Department of Medical Sciences, Ministry of Public Health
Mammals	
Human blood DNA	Faculty of Medicine, Chulalongkorn University (E.C. No. 654/60)^25^
Mouse DNA	Faculty of Medicine, Chulalongkorn University (E.C. No. 654/60)^25^

Strain number (e.g., ATCC15305) is not available for some samples, because DNA was isolated and sequencing identification was performed from local specimen.

For HPV positive controls and clinical samples, the respective genomic DNA were provided by the Buddhachinaraj Phitsanulok Hospital, Phitsanulok; and the Faculty of Medicine, Chulalongkorn University, Bangkok^23,27^. These sample collections and the protocols were approved by the Institutional Review Board of Buddhachinaraj Phitsanulok Hospital (101/54); and the Institutional Review Board of the Faculty of Medicine, Chulalongkorn University (COA No.278/2019, IRB No. 042/60), and the Institutional Biosafety Committee (IBC) of the Faculty of Medicine, Chulalongkorn University (MDCU-IBC016/2019), respectively. In brief, cervical swab samples were collected by genecologists from patients who visited the Buddhachinaraj Phitsanulok Hospital during 2013–2014 and the Department of Gynecological Out Patient, King Chulalongkorn Memorial Hospital, Bangkok, during 2019–2020. The samples were lysed in Pathogen Lysis Tubes L (Qiagen, Germany) using Tissue Lyser LT (Qiagen), and total nucleic acids were extracted automatically using the Cobas 4800 system (Roche Diagnostics). Then, these genomic DNA were HPV genotyped following the Cobas 4800 HPV test (Roche Diagnostics) and the REBA HPV-ID test (Molecules and Diagnostics); and were also the respective genomic DNA for our mRPA assay. All subjects provided written informed consent, and all data were fully anonymized before assessment. The study was performed in compliance with the Declaration of Helsinki.

### Optimization of mRPA reaction condition

The mRPA reaction (25 μL) comprised 0.12–0.48 µM each of primers (unless specified) in Table 1, the TwistAmp^®^ Basic reaction powder (TwistDx, Maidenhead, United Kingdom), 14.75 μL dehydration buffer (TwistDx), ~ 25 ng DNA (unless specified), and 1.25 μL of 280 mM magnesium acetate (pipetted inside the lid and spun down in the final mix step). The reaction was incubated at 39 °C (unless specified) for 40 min (unless specified). The optimization of the mRPA reaction condition included the determination of each minimal primer concentration that yielded both L1 and E6/E7 gene amplification products, and the shortest incubation time with the highest limit of detection (LOD) and correct specificity. The reaction was terminated by 80 °C heating for 2 min. The result was analyzed via 3% agarose gel electrophoresis: the amplicon sizes for L1 and E6/7 RPA were ~ 250 bp and ~ 170 bp, respectively.

### Evaluation of RPA primers by PCR and preparation of DNA templates

Single-gene PCR and L1-E6/E7 multiplex PCR (mPCR) were employed as the parallel nucleotide amplification experiments for our mRPA primers and LOD. The PCR reaction (25 µL) comprised 12.5 µL EmeraldAmp^®^ GT PCR Master Mix (TakaRa Bio, Shiga, Japan), 0.12–0.48 µM each primer, and ~ 25 ng DNA (unless specified). The PCR conditions were 95 °C 5 min followed by 35 cycles of 95 °C 15 s, 56 °C 30 s, and 72 °C 30 s, with a final extension at 72 °C 5 min. The proper size amplicon product was analyzed by 3% agarose gel electrophoresis. The amplicon sizes for L1 and E6/7 PCR were 188–194 bp and 117–121 bp, respectively. Quantitation of the amplicon was additionally performed using Qubit™ dsDNA HS and BR Assay Kits (Thermo Fisher Scientific, Massachusetts, USA). This kit quantifies the amount of amplicons (double-stranded DNA, dsDNA) at high sensitivity.

For preparation of HPV L1 (and E6/E7) gene as representative HPV HR and LR DNA templates, the PCR primers (Supplemental Table 1) flanking the RPA amplifying region templates were used. The PCR conditions were similar to the aforementioned, except for the annealing temperature and the product size as listed in Supplemental Table 1. The PCR products were agarose purified and measured quantity using Qubit™ dsDNA HS Assay Kit (Invitrogen). The copy number (copy/µL) was calculated using the following equation: (DNA concentration (ng/µL) × 6.0221 × 10^23^) ÷ (PCR product length (bp) × 6.6 × 10^11^).

### Comparison of HPV clinical sample diagnosis between our developed methods with conventional Cobas and REBA tests

To determine the performances of the conventional (Cobas and REBA) and our developed methods, statistically required sample numbers (N) (calculator.net/sample-size-calculator.html) were calculated according to the equation: N = (p (1-p) z^2^)/e^2^, given p at an average incidence of 75%, confidence level of 95%, and e of 7.5% for margin of error. This yielded an N of 129. Therefore, we tested 130 random HPV-positive and negative samples by our developed mRPA methods compared with the conventional Cobas and REBA results. The assay efficacy was calculated based on the following equations: sensitivity = true positive ÷ (true positive + false negative), specificity = true negative ÷ (true negative + false positive), false positive = 1 − specificity, false negative = 1 − sensitivity, and accuracy = (true positive + true negative) ÷ total samples.

## Results and discussion

### Specificity of mRPA

**Figure 1:**
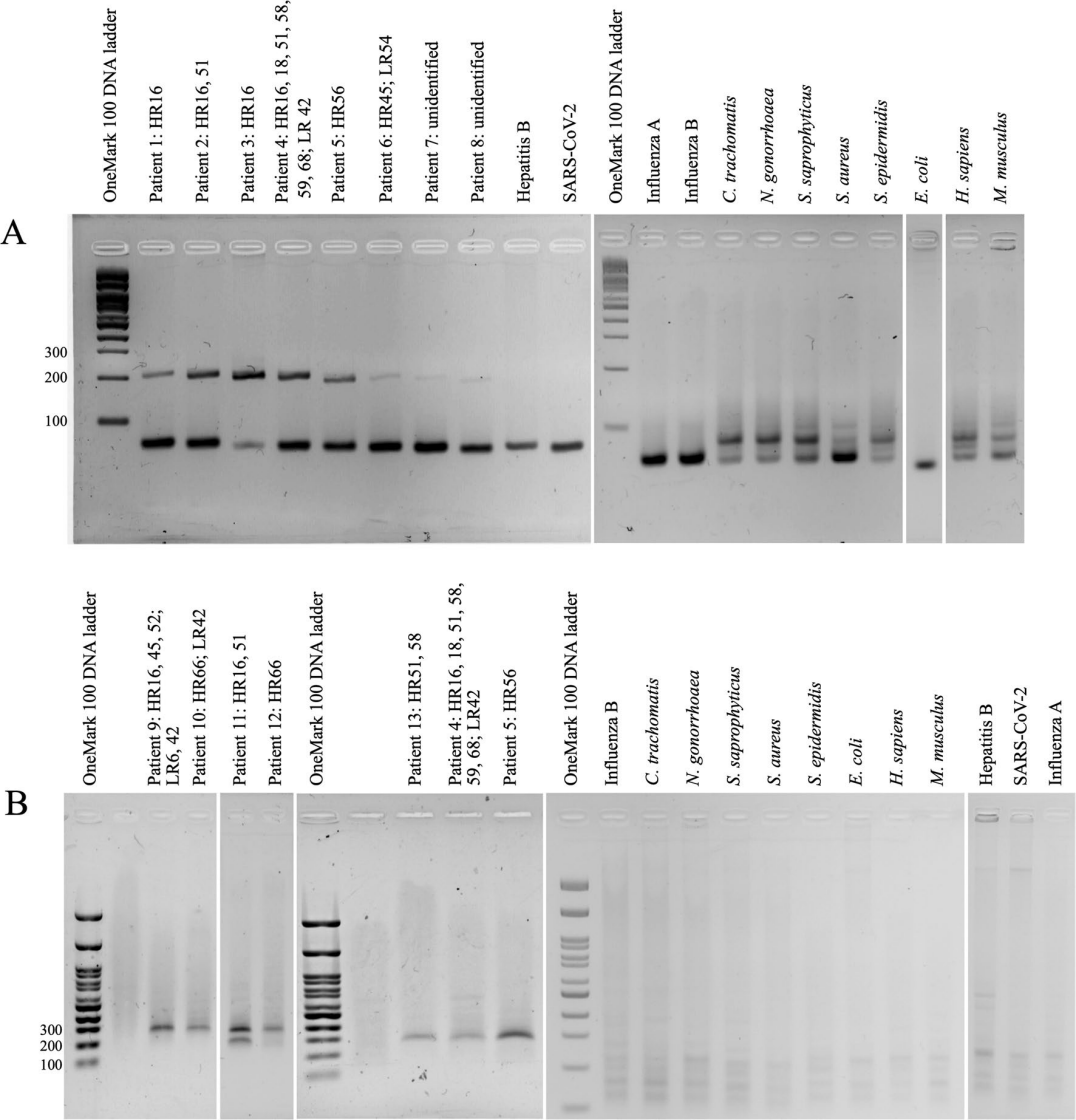
Specificity of our mRPA multiplex primers for L1 and E6/E7 genes by PCR (**A**) and RPA (**B**). Specificity assay included positive (HR types 16, 18, 45, 51, 52, 56, 58, 59, 62, 66; and LR type 6, 42, 54, 70) and negative (*C. trachomatis*, *N. gonorrhoeae*, *S. saprophyticus*, *S. aureus*, *S. epidermidis, E. coli*, hepatitis B, SARS-CoV-2, influenza A and B, *Homo sapiens*, *Mus musculus*, and sterile water) controls. Grouping of cropped gels were delineated with white spaces, and original uncropped images were in S1 File.

As sequences of HPV L1 and E6/E7 are highly variable among types, a primer multiplex system containing 8 degenerate primers was required to cover broad HPV L1 and E6/E7 gene types, of 20 HR (16, 18, 26, 31, 33, 34, 35, 39, 45, 51, 52, 53, 56, 58, 59, 66, 68, 69, 73, and 82) and 14 LR (6, 11, 32, 40, 42, 43, 44, 54, 61, 70, 72, 81, 84 and 87) types. We performed bioinformatic analyses by BLASTN on each HPV primer sequence specificity against NCBI GenBank non-redundant database (any other viruses, bacteria, mammals and plants), and the primers were specific to HPV (Table 1). Our primers, for example, RPA_L1_F showed no overlap of > 60% and no sequence identity of > 60% to any other viruses, bacteria, mammals and plants. The primers were then PCR checked for the specificity (Fig. 1A). We confirmed the specificity by individual gene RPA, and by mRPA, and the reactions were negative for all tested negative samples including sexually transmitted disease pathogens, urogenital normal flora, skin normal flora, viruses, and mammals (Fig. 1B). Noted that the mPCR and mRPA of the positive HPV HR samples showed only the L1 but not the E6/E7 gene band (only patients no. 11 and 12 showed E6/E6 RPA bands). We compared the %GC among primers and found that the RPA_E6/E7_F contains the relatively high %GC that might affect its higher melting temperature than the other primers and thus the harder to anneal to the template, causing the lower effective in E6/E7 than L1 amplification in mixed templates. Furthermore, in aspect of cancer progression when HPV genome integration occurs in > 80% of HPV-positive cervical cancers, the models of HPV DNA integration that promote oncogenesis known as E6/E7 super-enhancer disrupt the downstream E2 suppressor gene, where located the L2 and L1 genes, in orderly, to give a selective advantage. Breakpoints were often found within the early E1–E5 and late L1–L2 genes, and this inactivation of the E2 repressor upon HPV DNA integration is consistent with the enhanced E6/E7 expression^28,29^. Hence, the inclusion of E6/E7 in this study benefited the diagnosis in this HPV DNA integration subjects, and the mPCR and mRPA of the positive HPV HR samples that mostly no E6/E7 gene band appeared might reflect the non-precancerous state of the sample.

The RPA bands on the electrophoretic image were sometimes slightly skewed up ~ 100 bp might be because the unique auto-strand displacing mRPA amplicon conformations (with recombinase complex and single-stranded DNA binding protein (SSB) in RPA) making the amplicons heavier and we performed a simple one-step clean-up the reaction for low-cost and rapid potential local use, in replace of an amplicon purification step using commercial kits. The shifts of RPA amplicon bands had been reported elsewhere^30,31^. Noted that in our mPCR and mRPA displayed the < 100 bp bands (< ~ 200 bp for mRPA), which were primer dimers and non-specific amplification, and were found in some positive and negative clinical specificity samples, for examples, influenza B, *C. trachomatis*, *N. gonorrhoaea*, *S. saprophyticus*, *S. aureaus*, *S. epidermidis*, *H. sapiens* and *M. musculus* (Fig. 1).

### Optimal conditions and sensitivity (limit of detection) of mRPA

**Figure 2:**
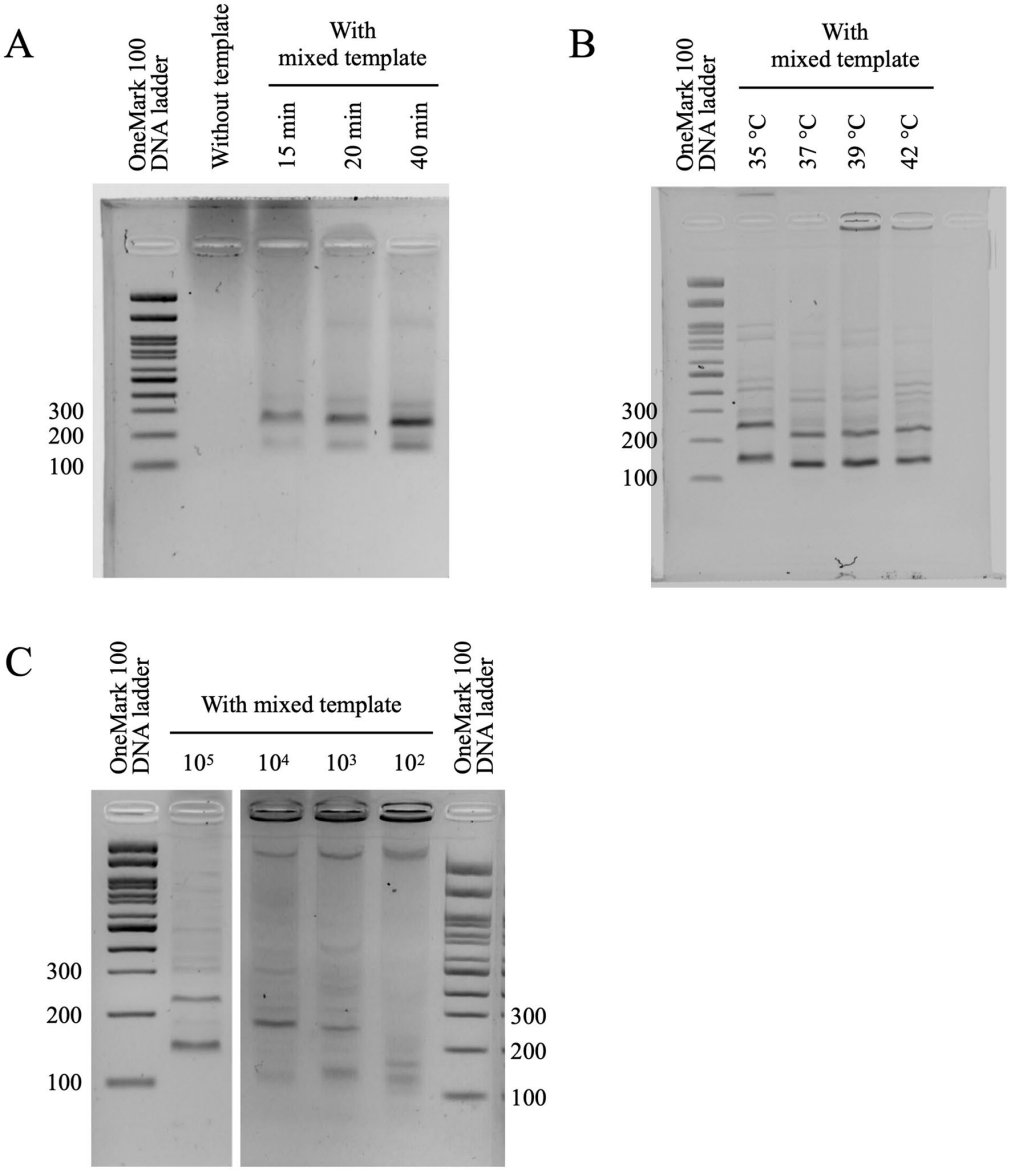
Agarose electrophoretic images of L1 and E6/E7 mRPA amplicons at optimal multiplex primer concentrations and incubation time (40 min) (**A**), incubation temperatures (**B**), and tenfold serial dilution determination of L1 and E6/E7 limits of detection (**C**), using mixed templates. In (**C**), grouping of cropped gels were delineated with white space, and original uncropped images were in S1 File.

We first attempted to use forward and reverse primers each at 0.48 µM concentration, but did not work for our mPCR and mRPA. Then, because we had two genes and one-to-more than one forward and reverse primers, we divided in approximate equal and started with 0.1, 0.2 0.3 and 0.4 µM each primer such that the overall concentration of each forward multiplex and each reverse multiplex primers were not much exceeding ~ 0.48 µM concentrations (the recommended concentration for TwistAmp™ Basic Kit). We found the minimal concentration of multiplex primers for L1 (0.1–0.2 µM each) and E6/E7 (0.2 µM each) RPA to be different (Table 3A, Supplemental Fig. 1). In continuing, we tested the L1 and E6/E7 mRPA using a different combination of both gene multiplex primer concentrations to determine the minimal concentration where the mRPA of both genes worked meanwhile the good range of limit of detection. We followed the multiplex primer adjusting methods by Sint et al.^32^ to obtain equal amplification efficiency. In brief, each gene primer was individually checked for a single amplification fragment, given that synthetic PCR amplicons of HR-HPV types 18 and 33 and LR-HPV type 11 also supported the validation of the effectiveness of the primers (Fig. 2, Supplemental Table 1). Then, the multiplex primers were tested from a gradient of low to higher concentrations with single and mixed templates to determine the optimal concentrations, and some primer concentrations were finally adjusted stepwise to obtain equal amplification efficiency by decreasing those that resulted in relatively strong signal and increasing the ones producing too weak bands in steps of ~ 0.1 µM. We found 0.12–0.24 µM each for the L1 primers and 0.24–0.48 µM each for the E6/E7 primers (Table 3A). For the optimal incubation time, although the amplicons were observed faintly since 15 min, the 40 min incubation allowed clear distinctiveness of both amplicons (Table 3B, Fig. 2A). For an incubation temperature, the RPA enzyme and reaction were suggested at 39 °C by TwistDx, and the optimal incubation temperatures of 35 °C, 37 °C and 42 °C were additionally performed to determine if incubation temperature adjustment was needed. We found that the optimal incubation temperature for mRPA could be 37 or 39 °C due to their relatively strongest bands (Fig. 2B). The limit of detection at the optimal conditions of mRPA was found at 1,000 copies (equivalent to 2.01 fg) for L1 and 100 copies (0.0125 fg) for E6/E7 (Fig. 2C: a faint band of E6/E7 at ~ 170 bp). For clear and confirmatory LOD determination of E6/E7, the mRPA reaction was also tested with a single E6/E7 template and the faint band remained observed at 100 copies (Supplemental Fig. 2). These limits of detection were comparable to some conventional assays, i.e. 1000 copies LOD by Human Papilloma Virus (HPV) DNA Diagnostic Kit (3DMed Diagnostics, Shanghai, China) and 10,000 copies by Primary Screening HPV Test Kit (Jiangsu Mole Bioscience, Jiangsu, China). The obstacles of the lower sensitivity and specificity were often reported for multiplex primers yet to replace with the powerful and cost-effective simple single-tube HPV HR and LR mRPA that is still very critical for the screening purpose in many clinical and epidemiology settings^33,34^.

**Table 3:** Optimal conditions for mRPA primer concentrations (A), and incubation time (B).

Multiplex primer concentration		RPA (1 gene)	mRPA (2 genes)		
A					
L1	0.2 µM RPA_L1_F 0.1 µM RPA_L1_R1 0.1 µM RPA_L1_R2	✓	✗		
	0.24 µM RPA_L1_F 0.12 µM RPA_L1_R1 0.12 µM RPA_L1_R2	✓	✓		
	0.4 µM RPA_L1_F 0.2 µM RPA_L1_R1 0.2 µM RPA_L1_R2	✓	✓		
	0.6 µM RPA_L1_F 0.3 µM RPA_L1_R1 0.3 µM RPA_L1_R2	✓	✓		
E6/E7	0.1 µM RPA_E6/E7_F 0.1 µM RPA_ E6/E7_R1 0.1 µM RPA_ E6/E7_R2 0.1 µM RPA_ E6/E7_R3 0.1 µM RPA_ E6/E7_R4	✗	✗		
	0.2 µM RPA_ E6/E7_F 0.2 µM RPA_ E6/E7_R1 0.2 µM RPA_ E6/E7_R2 0.2 µM RPA_ E6/E7_R3 0.2 µM RPA_ E6/E7_R4	✓	✗		
	0.3 µM RPA_ E6/E7_F 0.3 µM RPA_ E6/E7_R1 0.3 µM RPA_ E6/E7_R2 0.3 µM RPA_ E6/E7_R3 0.3 µM RPA_ E6/E7_R4	✓	✗		
	0.4 µM RPA_ E6/E7_F 0.4 µM RPA_ E6/E7_R1 0.4 µM RPA_ E6/E7_R2 0.4 µM RPA_ E6/E7_R3 0.4 µM RPA_ E6/E7_R4	✓	✗		
	0.4 µM RPA_ E6/E7_F 0.2 µM RPA_ E6/E7_R1 0.2 µM RPA_ E6/E7_R2 0.2 µM RPA_ E6/E7_R3 0.2 µM RPA_ E6/E7_R4	✓	✗		
	0.4 µM RPA_ E6/E7_F 0.2 µM RPA_ E6/E7_R1 0.2 µM RPA_ E6/E7_R2 0.2 µM RPA_ E6/E7_R3 0.4 µM RPA_ E6/E7_R4	✓	✗		
	0.48 µM RPA_ E6/E7_F 0.24 µM RPA_ E6/E7_R1 0.24 µM RPA_ E6/E7_R2 0.24 µM RPA_ E6/E7_R3 0.48 µM RPA_ E6/E7_R4	✓	✓		

Time (min)	10	15	20	40	60
B					
0.24 µM RPA_L1_F 0.12 µM RPA_L1_R1 0.12 µM RPA_L1_R2 0.48 µM RPA_E6/E7_F 0.24 µM RPA_ E6/E7_R1 0.24 µM RPA_ E6/E7_R2 0.24 µM RPA_ E6/E7_R3 0.48 µM RPA_ E6/E7_R4	✗	✓	✓	✓	✓

**Figure 3:**
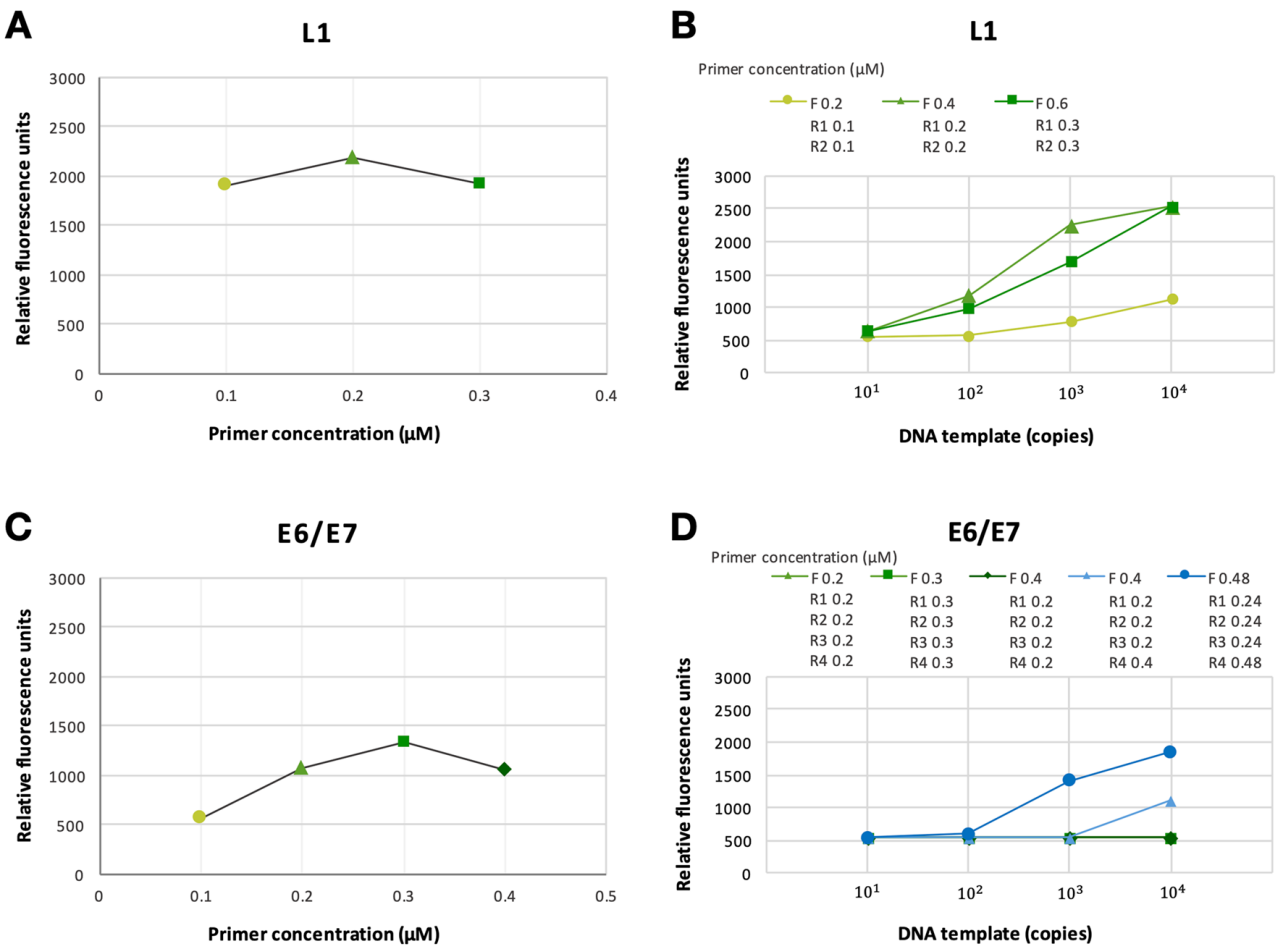
Fluorescent quantitation to validate optimal range of primer concentrations in 1-gene PCR reactions (**A** and **C**, L1 and E6/E7) and multiplex 2-gene PCR reactions (**B** and **D**). In (**A** and **C**), individual primers were added at specified 0.1–0.4 µM on X-axis and synthetic L1 (or E6/E7) amplicons at > 10^8^ copies were used as template. For (**B** and **D**), primers were added following primer concentration selections from Table 3 and synthetic L1 (or E6/E7) amplicons at 10^1^, 10^2^, 10^3^ and 10^4^ copies were used as template.

To confirm the color depth of the band, we quantified the amount of amplicons at high sensitivity with double-stranded DNA fluorescent dye using Qubit™ dsDNA HS and BR Assay Kits (Thermo Fisher Scientific). Individual L1 (and E6/E7) primers could successfully amplified the HPV templates (Fig. 3A,C). Consistent with the semi-quantitative agarose electrophoretic analyses (Table 3), the fluorescent quantitation of the optimal mRPA primer concentrations demonstrated the relatively greatest fluorescent units (Fig. 3B,D).

### Comparison of HPV clinical sample diagnosis of our developed methods with conventional Cobas and REBA tests

**Figure 4:**
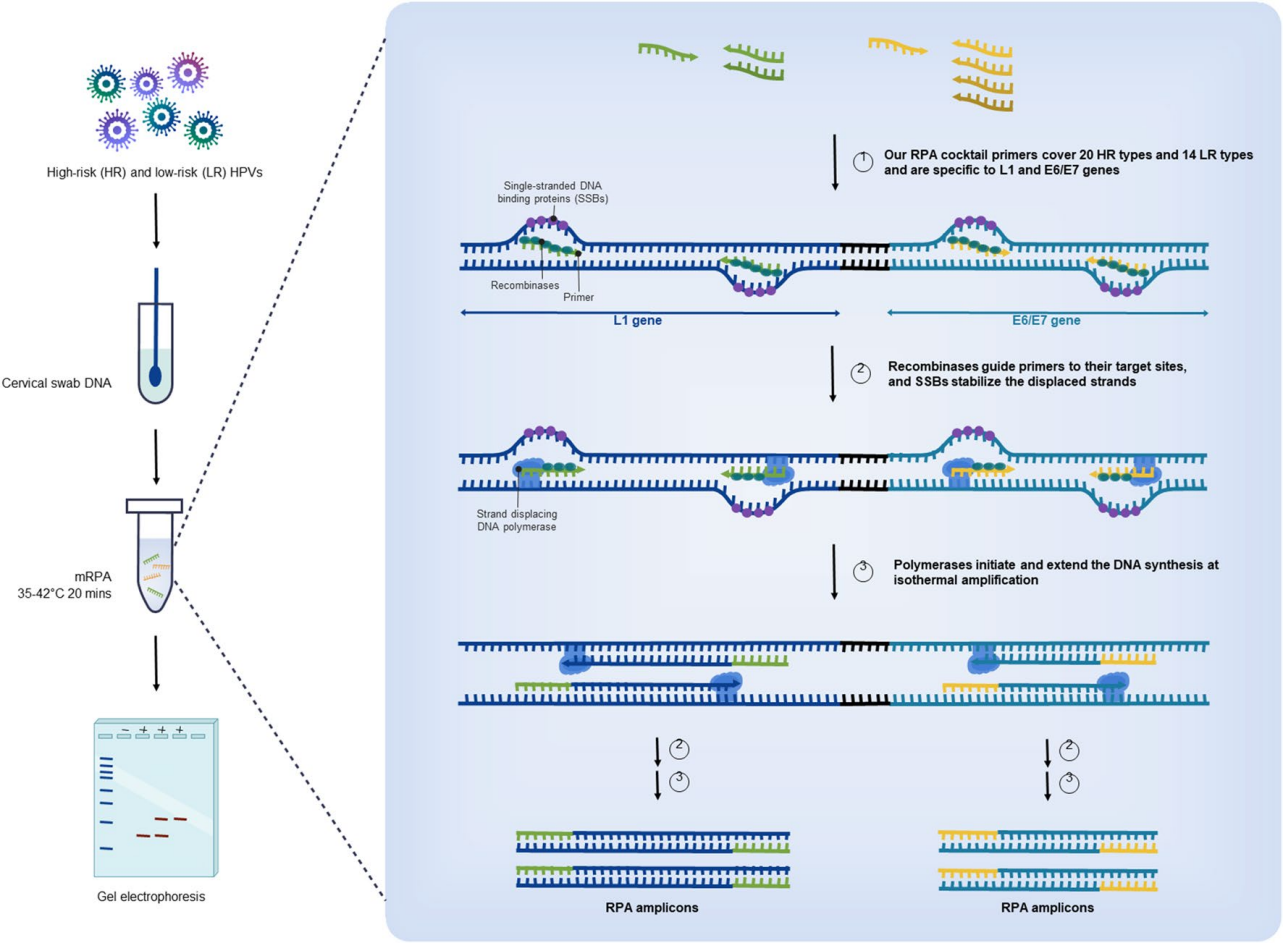
Schematic illustration of RPA and multiplex 2-gene amplification concepts for potential local applications.

The schematic illustration of RPA and multiplex 2-gene amplification concepts as local HR- and LR-HPV screening tool was summarized in Fig. 4. For potential local use of our single-tube mRPA as broad HPV test (20 HR and 14 LR types) in 40 min, we evaluated the feasibility and compared the assay performance on a statistical number of real clinical DNA samples (N = 130 samples), with Cobas and REBA results. Our developed mRPA assay demonstrated 72–100% specificity, 71–75% sensitivity and 72–78% accuracy, with < 1 h assay time (20–40 min mRPA and 20 min agarose gel electrophoresis) and 2.3–6.7 folds lower price per reaction (Table 4, S2 File). The approximately 75% assay accuracy and 100–10,000 limit of detection are in the reported ranges of other HPV assays and could still serve useful as HPV screening, especially in resource-constrained settings as the reaction requires no specific equipment^35,36^. Meanwhile, the Cobas and REBA results exhibited 18.46% diagnostic disagreement between each other. Furthermore, the mRPA result analysis by agarose gel electrophoresis may be replaced by using fluorescent DNA dye, such as SYBR Green I (Invitrogen, New York, USA), or DNA probe paper strip for an even more rapid total assay time. We are continuing to improve the mRPA limit of detection by reducing the number of primers to focus on the HPV HR types and the gene expression detection of E6/E7 as reverse transcription-mRPA^37^.

**Table 4:** Comparison of our developed assay (mRPA) to conventional diagnostic assays (Cobas and REBA) on 130 clinical DNA samples.

mRPA	Cobas		REBA	
	HPV positive	HPV positive	HPV negative	HPV positive
Positive	True positive = 75	False positive = 7	True positive = 87	False positive = 0
Negative	False negative = 30	True negative = 18	False negative = 29	True negative = 14
Specificity (%)	72		100	
Sensitivity (%)	71		75	
False positive (%)	15		0	
False negative (%)	37		33	
Accuracy (%)	72		78	
Assay time	mRPA < 1 h/ Cobas 5 h/ REBA 6 h			
Price per reaction	mRPA 3.68 USD/Cobas 8.42 USD/REBA 24.86 USD			

## Conclusions

Our developed HPV mRPA assay for broad-type HPV detection (Thai patent pending entitled multiplex recombinase polymerase amplification assay for detection of high risk type and low risk type HPV, with ease, rapidity, low cost, high sensitivity and specificity, in a single tube) is simple, rapid, inexpensive, specific, sensitive, and does not require any complicated instrumentation (incubation may be accomplished by hand holding or appropriate tropical temperature may be possible) (Fig. 4). Evaluation of the assay over positive–negative controls showed proper detections, and on 130 clinical samples compared with the conventional commercial Cobas and REBA assays, both showing 18.46% result disagreement, showed greater than 70% assay effectiveness (72–78% accuracy). The detection limit is as low as 1000 copies (2.01 fg) of the L1 HR and LR types and 100 copies (0.0125 fg) of the E6/E7, and the developed assay could detect at least 20 HPV HR and 14 LR types. Further, the reaction incubation might be performed via hand holding and the alternative fluorescent DNA dye may be used to replace the agarose gel electrophoresis. This developed assay is, thereby, a promising tool for potential screening in local and low-resource settings, to allow disease detection before the cancerous state of the disease.

## Supplementary Information

Supplementary Information 1.

Supplementary Information 2.

Supplementary Information 3.

## Data Availability

All data generated or analysed during this study are included in this published article and its supplementary information files.

## References

[1] Stelzle D, Tanaka LF, Lee KK, Khalil AI, Baussano I, Shah ASV (2020). Estimates of the global burden of cervical cancer associated with HIV. Lancet Glob. Health.

[2] Sung H, Ferlay J, Siegel RL, Laversanne M, Soerjomataram I, Jemal A (2021). Global cancer statistics 2020: GLOBOCAN estimates of incidence and mortality worldwide for 36 cancers in 185 countries. CA Cancer J. Clin..

[3] ICO/IARC (Institute of Oncology/ International Agency for Research on Cancer) Information Centre on HPV and Cancer. HPV Information Centre. Thailand human papillomavirus and related cancers, fact sheet 2021. https://hpvcentre.net/statistics/reports/THA_FS.pdf.

[4] Katanyoo K, Riewpaiboon A, Chaikledkaew U, Thavorncharoensap M (2021). The cost of locally advanced cervical cancer in Thailand: An empirical study for economic analysis. Asian Pac. J. Cancer Prev..

[5] Muñoz N, Bosch FX, de Sanjosé S, Herrero R, Castellsagué X, Shah KV (2003). Epidemiologic classification of human papillomavirus types associated with cervical cancer. N. Engl. J. Med..

[6] Smith JS, Lindsay L, Hoots B, Keys J, Franceschi S, Winer R (2007). Human papillomavirus type distribution in invasive cervical cancer and high-grade cervical lesions: A meta-analysis update. Int. J. Cancer.

[7] Combes JD, Franceschi S (2014). Role of human papillomavirus in non-oropharyngeal head and neck cancers. Oral Oncol..

[8] Burd EM (2003). Human papillomavirus and cervical cancer. Clin. Microbiol. Rev..

[9] Vo DT, Story MD (2021). Facile and direct detection of human papillomavirus (HPV) DNA in cells using loop-mediated isothermal amplification (LAMP). Mol. Cell Probes.

[10] Mayrand MH, Duarte-Franco E, Rodrigues I, Walter SD, Hanley J, Ferenczy A (2007). Human papillomavirus DNA versus Papanicolaou screening tests for cervical cancer. N. Engl. J. Med..

[11] Boone JD, Erickson BK, Huh WK (2012). New insights into cervical cancer screening. J. Gynecol. Oncol..

[12] Spence AR, Goggin P, Franco EL (2007). Process of care failures in invasive cervical cancer: Systematic review and meta-analysis. Prev. Med..

[13] Ronco G, Dillner J, Elfstrom KM, Tunesi S, Snijders PJ, Arbyn M (2014). Efficacy of HPV-based screening for prevention of invasive cervical cancer: Follow-up of four European randomised controlled trials. Lancet.

[14] Vink MA, Bogaards JA, Meijer CJ, Berkhof J (2015). Primary human papillomavirus DNA screening for cervical cancer prevention: Can the screening interval be safely extended?. Int. J. Cancer.

[15] Liu SS, Chan KKL, Wei TN, Tse KY, Ngu SF, Chu MMY (2022). Clinical performance of the Roche Cobas 4800 HPV test for primary cervical cancer screening in a Chinese population. PLoS ONE.

[16] Kim S, Lee D, Park S, Kim TU, Jeon B-Y, Park KH (2012). REBA HPV-ID for efficient genotyping of human papillomavirus in clinical samples from Korean patients. J. Med. Virol..

[17] Amer HM, Wahed AAE, Shalaby MA, Almajhdi FN, Hufert FT, Weidmann M (2013). A new approach for diagnosis of bovine coronavirus using a reverse transcription recombinase polymerase amplification assay. J. Virol. Methods.

[18] Ma B, Fang J, Wang Y, He H, Dai M, Lin W (2017). Isothermal method of a recombinase polymerase amplification assay for the detection of most common high-risk human papillomavirus type 16 and type 18 DNA. Clin. Lab..

[19] Gong J, Zhang G, Wang W (2021). A simple and rapid diagnostic method for 13 types of high-risk human papillomavirus (HR-HPV) detection using CRISPR-Cas12a technology. Sci. Rep..

[20] Pal A, Kundu R (2020). Human papillomavirus E6 and E7: the cervical cancer hallmarks and targets for therapy. Front. Microbiol..

[21] Ye J, Coulouris G, Zaretskaya I, Cutcutache I, Rozen S, Madden TL (2012). Primer-BLAST: A tool to design target-specific primers for polymerase chain reaction. BMC Bioinform..

[22] Higgins M, Ravenhall M, Ward D, Phelan J, Ibrahim A, Forrest MS (2019). PrimedRPA: Primer design for recombinase polymerase amplification assays. Bioinformatics.

[23] Somboonna N, Choopara I, Arunrut N, Sukhonpan K, Sayasathid S, Dean D (2018). Rapid and sensitive detection of *Chlamydia trachomatis* sexually transmitted infections in resource-constrained settings in Thailand at the point-of-care. PLoS Negl. Trop. Dis..

[24] Srimongkol G, Ditmangklo B, Choopara I, Thaniyavarn J, Dean D, Kokpol S (2020). Rapid colorimetric loop-mediated isothermal amplification for hypersensitive point-of-care *Staphylococcus aureus* enterotoxin A gene detection in milk and pork products. Sci. Rep..

[25] Choopara I, Suea-Ngam A, Howes PD, Schmelcher M, Leelahavanichkul A, Thunyaharn S (2021). Fluorometric paper-based loop-mediated isothermal amplification (LAMP) device for quantitative point-of-care detection of methicillin resistant *Staphylococcus aureus* (MRSA). ACS Sens..

[26] Choopara I, Teethaisong Y, Aunrut N, Thunyaharn S, Kiatpathomchai W, Somboonna N (2021). Specific and sensitive, ready-to-use universal fungi detection by visual color using ITS1 loop-mediated isothermal amplification combined hydroxynaphthol blue. PeerJ.

[27] Somboonna N, Choopara I, Sukhonpan K, Sayasathid J (2015). Sexually transmitted diseases in symptomatic and asymptomatic Thai females (a study from Bangkok and vicinity). Asian Biomed..

[28] McBride AA, Warburton A (2017). The role of integration in oncogenic progression of HPV-associated cancers. PLoS Pathog..

[29] Holmes A, Laneiras S, Jeannot E, Marie Y, Castera L, Sastre-Garau X (2016). Mechanistic signatures of HPV insertions in cervical carcinomas. npj Genomic Med..

[30] Georgoutsou-Spyridonos M, Filippidou M, Kaprou GD, Mastellos DC, Chatzandroulis S, Tserepi A (2021). Isothermal recombinase polymerase amplification (RPA) of *E. coli* gDNA in commercially fabricated PCB-based microfluidic platforms. Micromachines.

[31] Wang H, Hou P, Zhao G, Yu L, Gao Y-W, He H (2018). Development and evaluation of serotype-specific recombinase polymerase amplification combined with lateral flow dipstick assays for the diagnosis of foot-and-mouth disease virus serotype A, O and Asia1. BMC Vet. Res..

[32] Sint D, Raso L, Traugott M (2012). Advances in multiplex PCR: Balancing primer efficiencies and improving detection success. Methods Ecol. Evol..

[33] Elnifro EM, Ashshi AM, Cooper RJ, Klapper PE (2000). Multiplex PCR: Optimization and application in diagnostic virology. Clin. Microbiol. Rev..

[34] Li J, Pollak NM, MacDonald J (2019). Multiplex detection of nucleic acids using recombinase polymerase amplification and a molecular colorimetric 7-segment display. ACS Omiga.

[35] Hesselink AT, Berkhof J, van der Salm ML, van Splunter AP, Geelen TH, van Kemenade FJ (2014). Clinical validation of the HPV-risk assay, a novel real-time PCR assay for detection of high-risk human papillomavirus DNA by targeting the E7 region. J. Clin. Microbiol..

[36] Ozaki S, Kato K, Abe Y, Hara H, Kubota H, Kubushiro K (2014). Analytical performance of newly developed multiplex human papillomavirus genotyping assay using Luminex xMAP™ technology (Mebgen™ HPV Kit). J. Virol. Methods.

[37] Weston G, Dombrowski C, Harvey MJ, Iftner T, Kyrjiou M (2020). Use of the Aptima mRNA high-risk human papillomavirus (HR-HPV) assay compared to a DNA HR-HPV assay in the English cervical screening programme: A decision tree model based economic evaluation. BMJ Open.

