# Supplementary material for: Multiplex recombinase polymerase amplification for high-risk and low-risk type HPV detection, as potential local use in single tube
Source: Sci Rep. 2023 Jan 16;13:829. doi: 10.1038/s41598-023-28038-9 (PMC9841928; doi:10.1038/s41598-023-28038-9)
Supplement: Supplementary file 1 — Supplementary Information 1. [file 41598_2023_28038_MOESM1_ESM.pdf]

S1 File. Original uncropped and unadjusted gel electrophoresis images of Figures 1 and 2C. The other figures are of original images.

Fig 1A

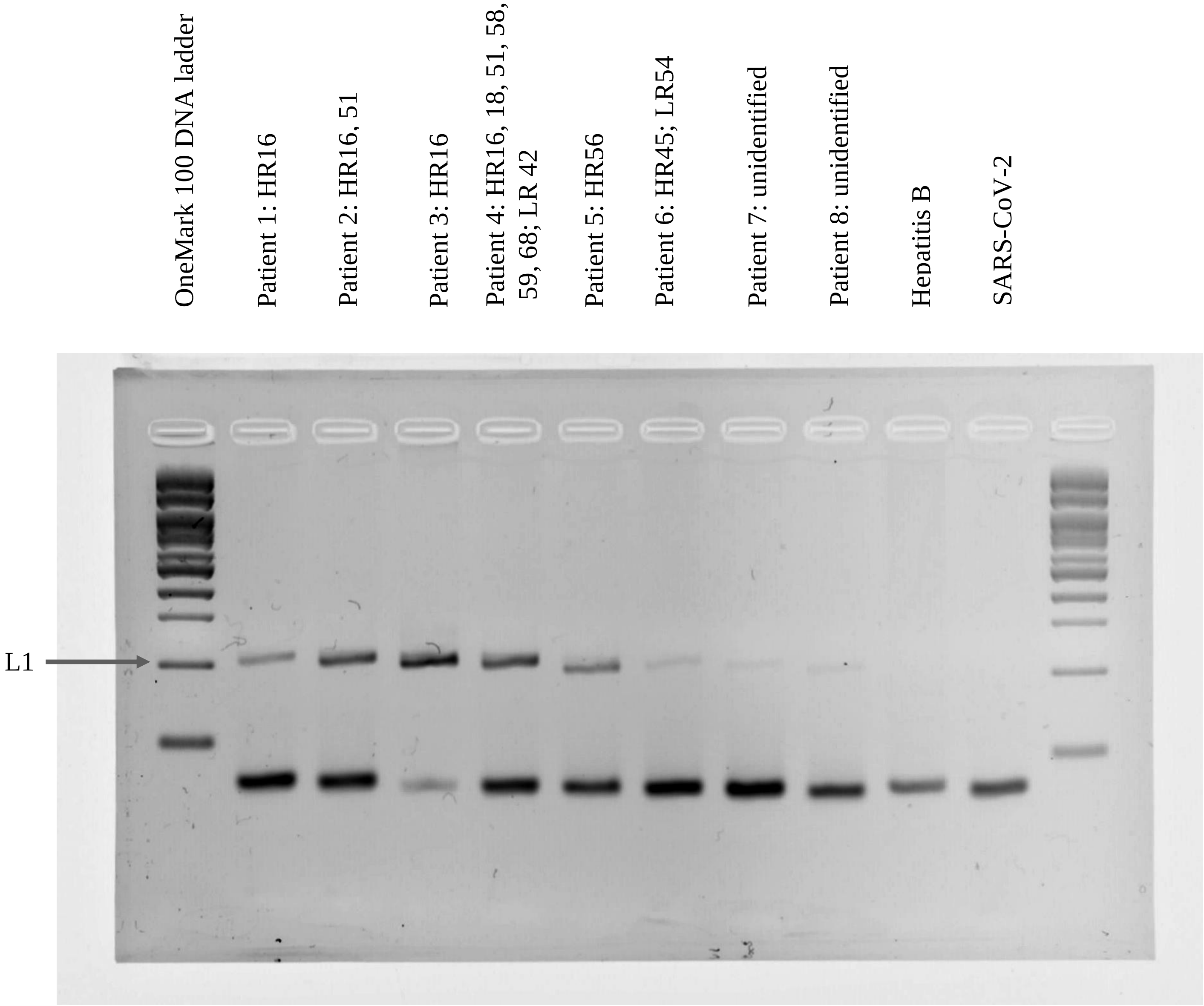
OneMark 100 DNA ladder
Patient 1: HR16
Patient 2: HR16, 51
Patient 3: HR16
Patient 4: HR16, 18, 51, 58, 59, 68; LR 42
Patient 5: HR56
Patient 6: HR45; LR54
Patient 7: unidentified
Patient 8: unidentified
Hepatitis B
SARS-CoV-2
L1

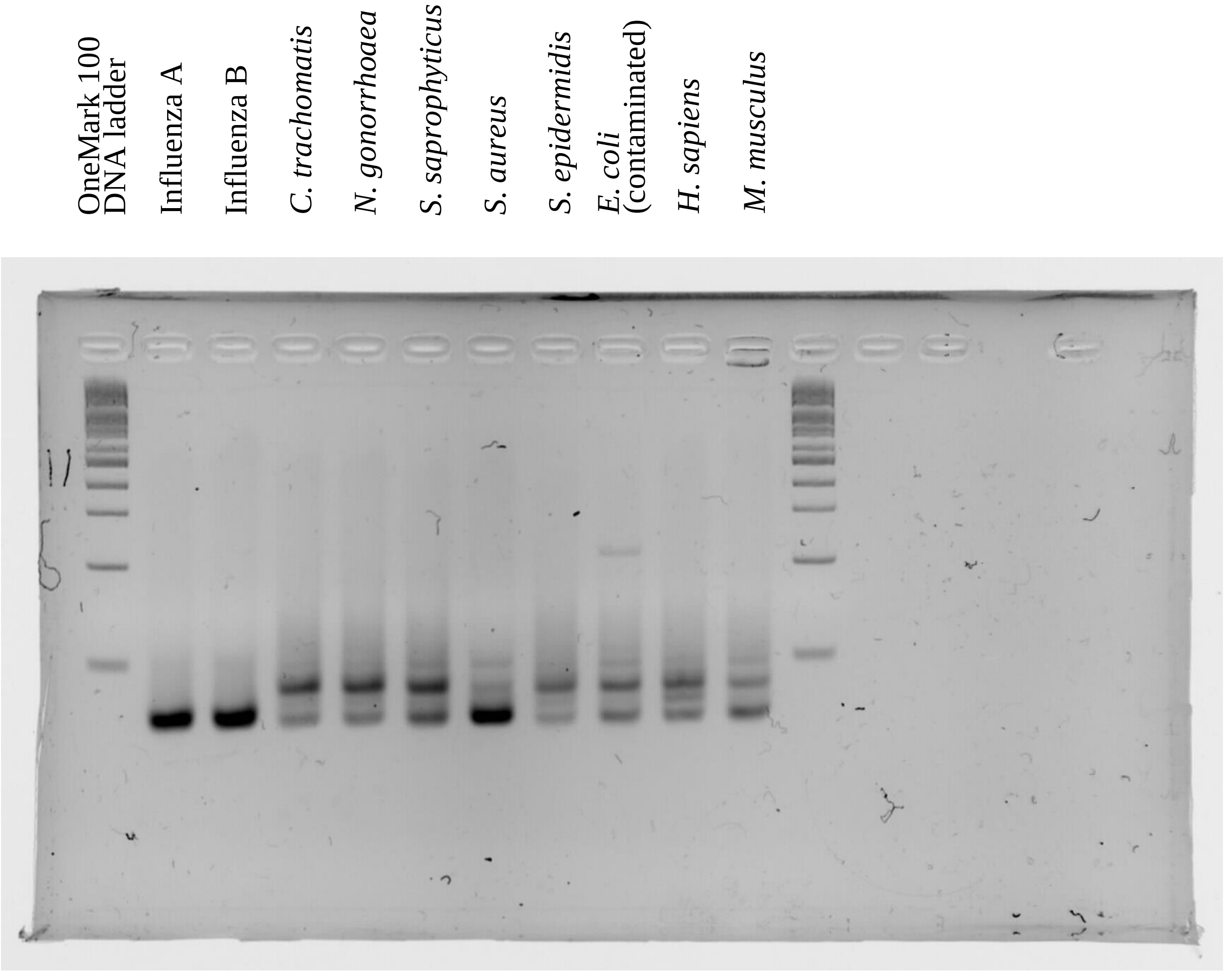

OneMark 100 DNA ladder
Influenza A
Influenza B
C. trachomatis
N. gonorrhoaea
S. saprophyticus
S. aureus
S. epidermidis
E. coli (contaminated)
H. sapiens
M. musculus

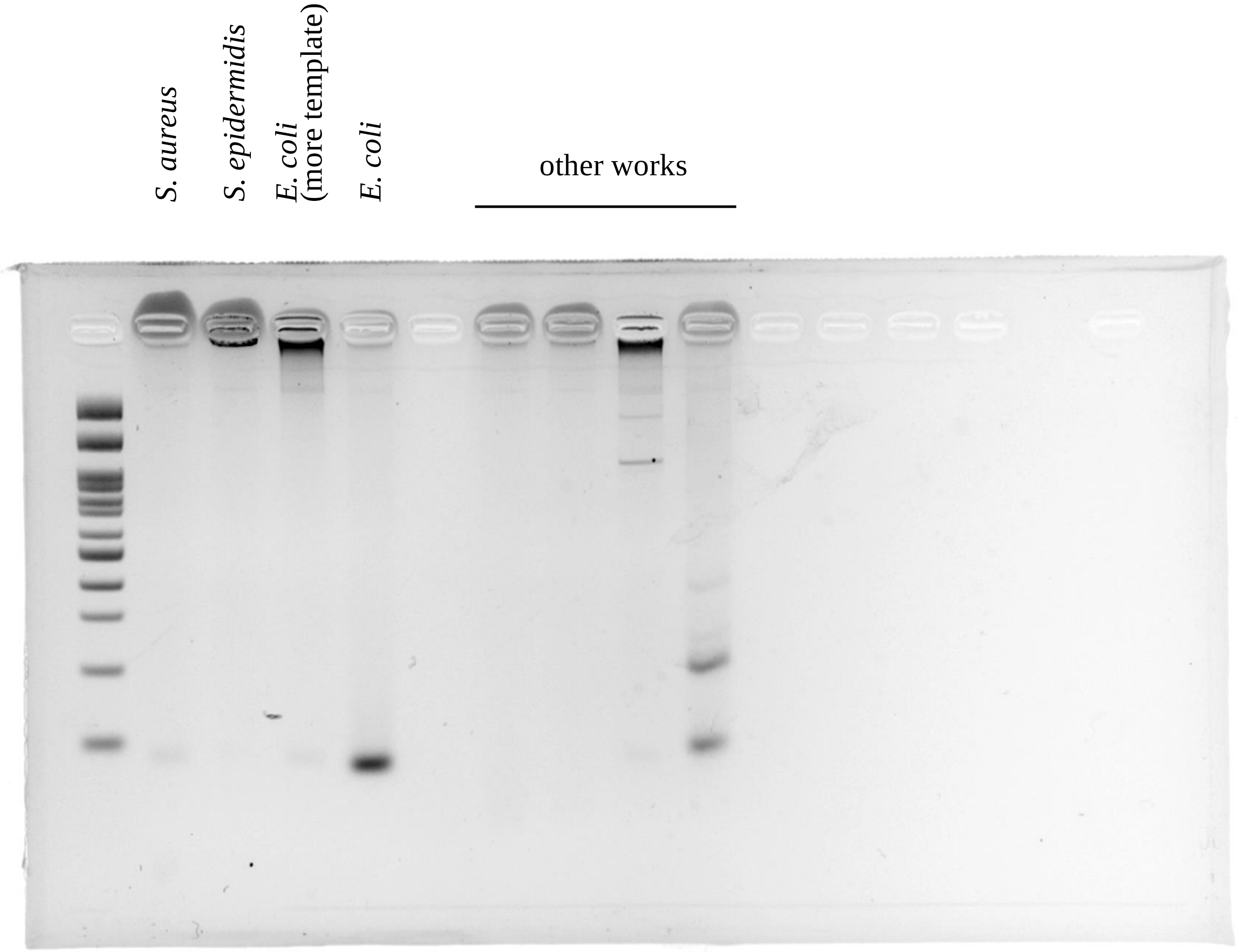
S. aureus
S. epidermidis
E. coli
(more template)
E. coli
other works

Fig 1B

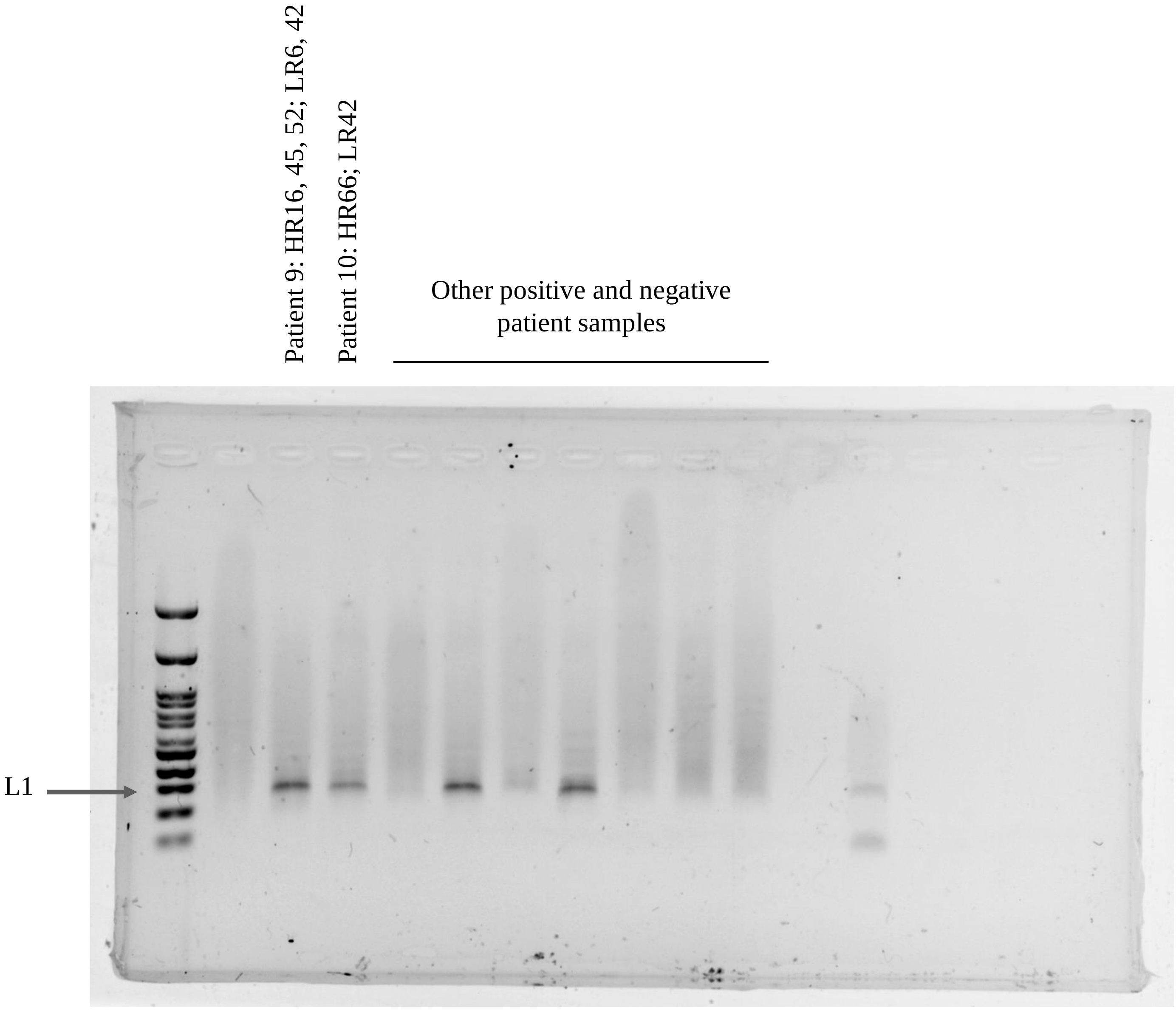
Patient 9: HR16, 45, 52; LR6, 42
Patient 10: HR66; LR42
Other positive and negative patient samples
L1

Other positive and negative patient samples

Patient 11: HR16, 51

Patient 12: HR66

Other positive and negative patient samples

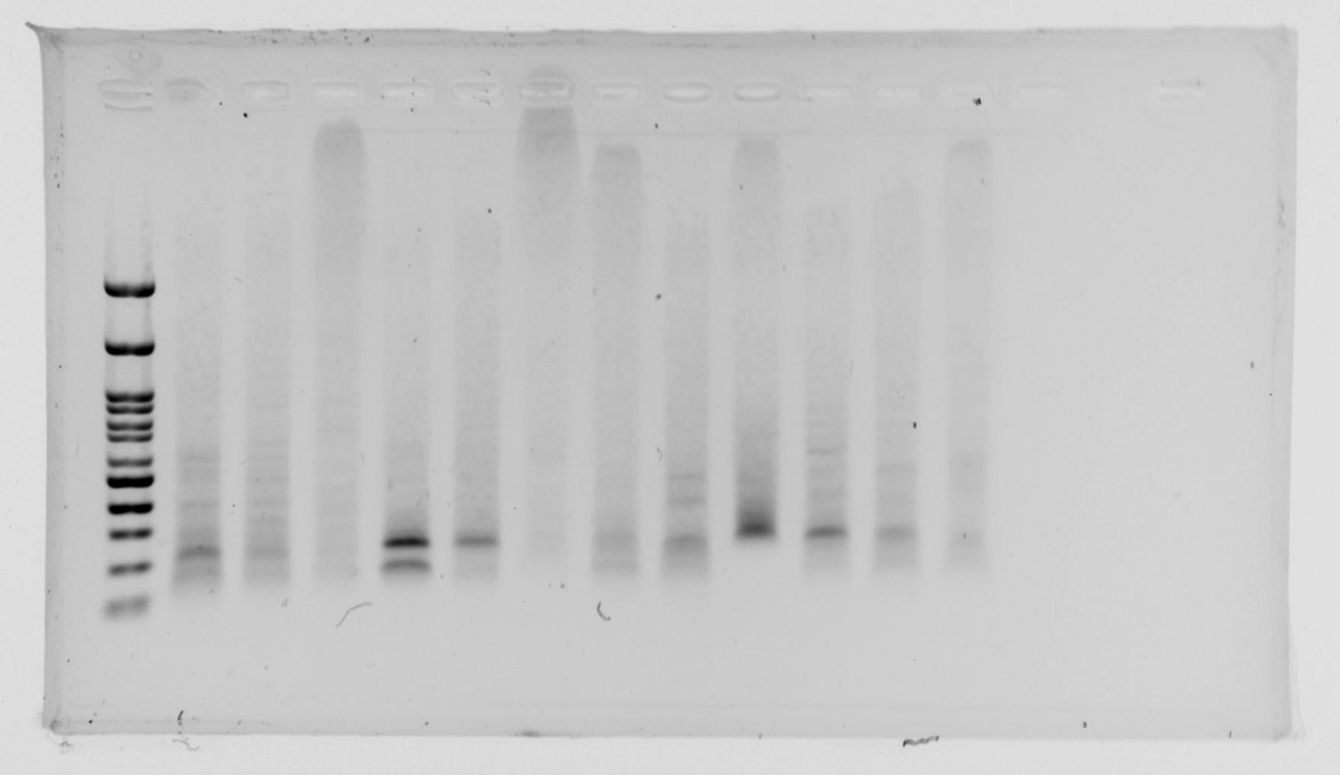

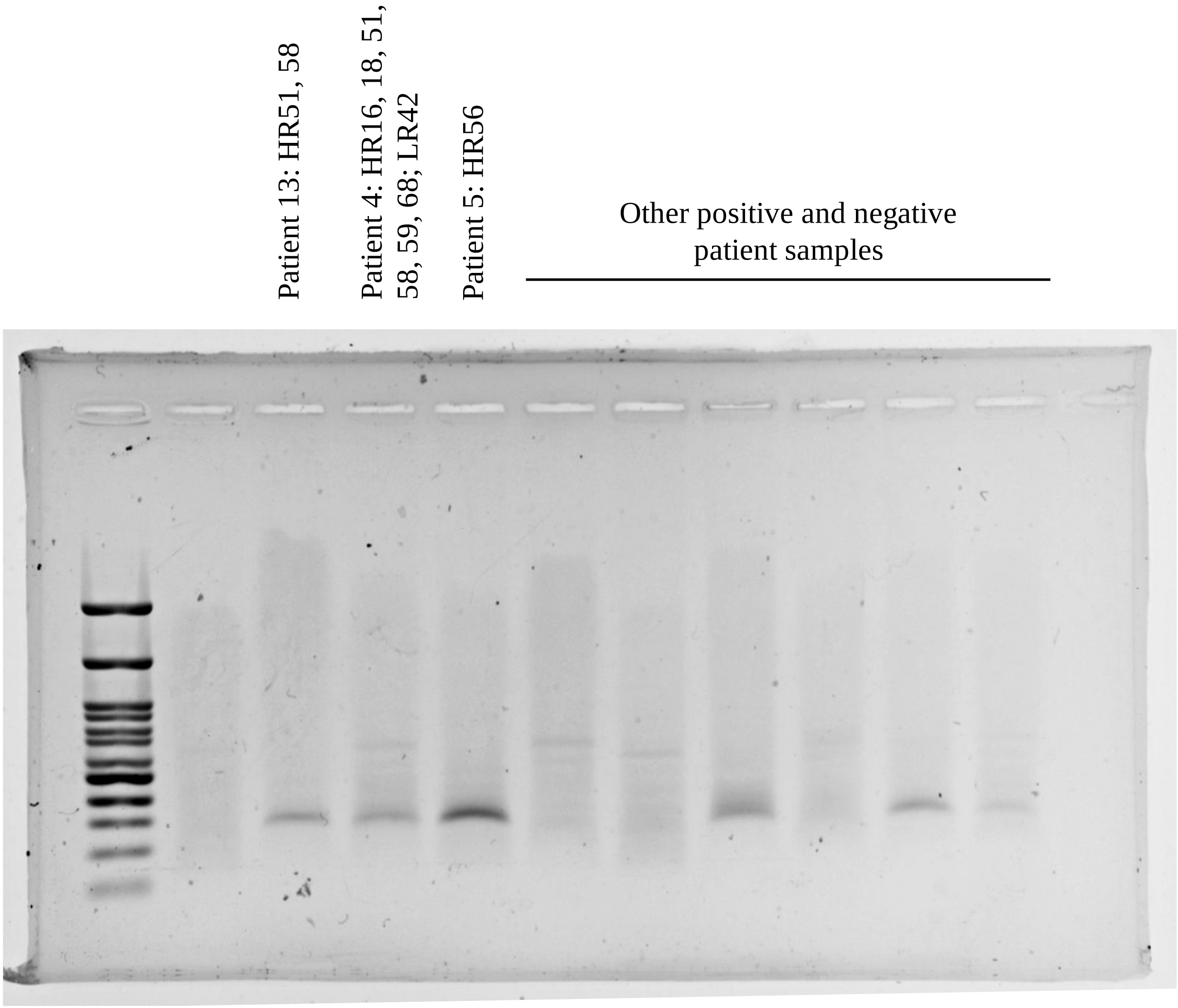
Patient 13: HR51, 58
Patient 4: HR16, 18, 51, 58, 59, 68; LR42
Patient 5: HR56
Other positive and negative patient samples

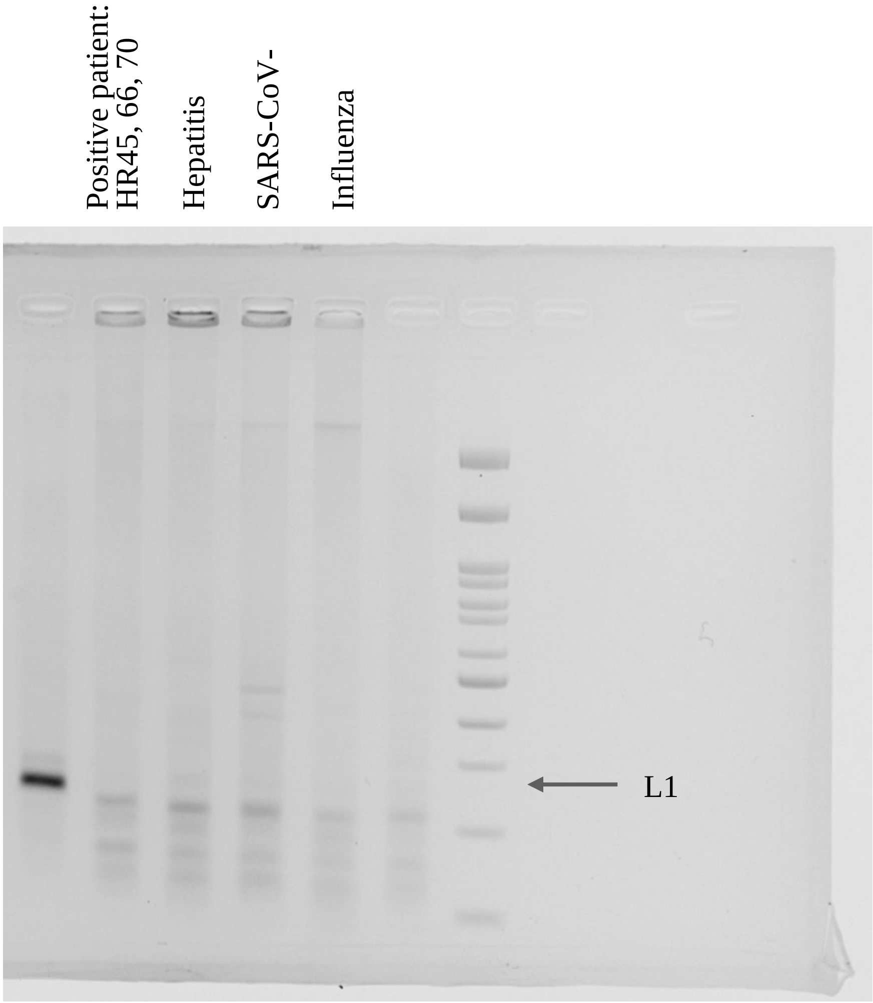

Positive patient: HR45, 66, 70
Hepatitis
SARS-CoV-
Influenza
L1

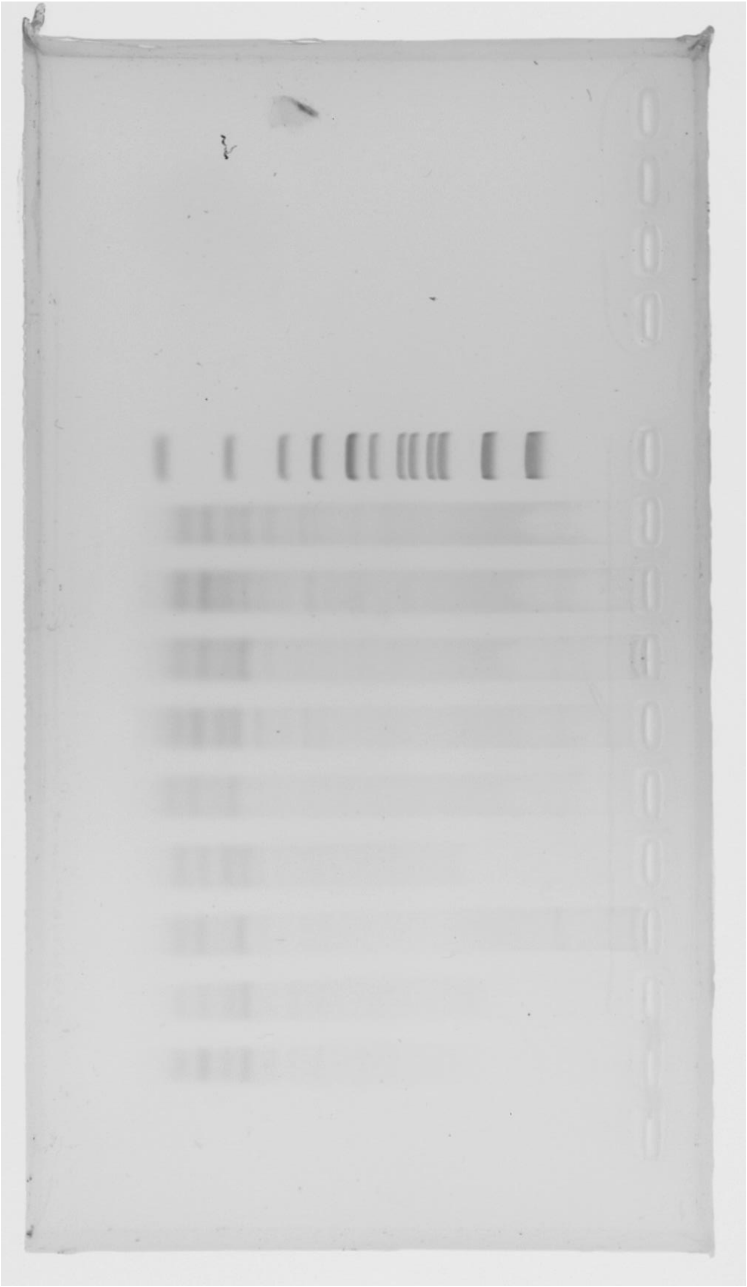

Influenza B
C. trachomatis
N. gonorrhoaea
S. saprophyticus
S. aureus
S. epidermidis
E. coli
H. sapiens
M. musculus

Fig 2C

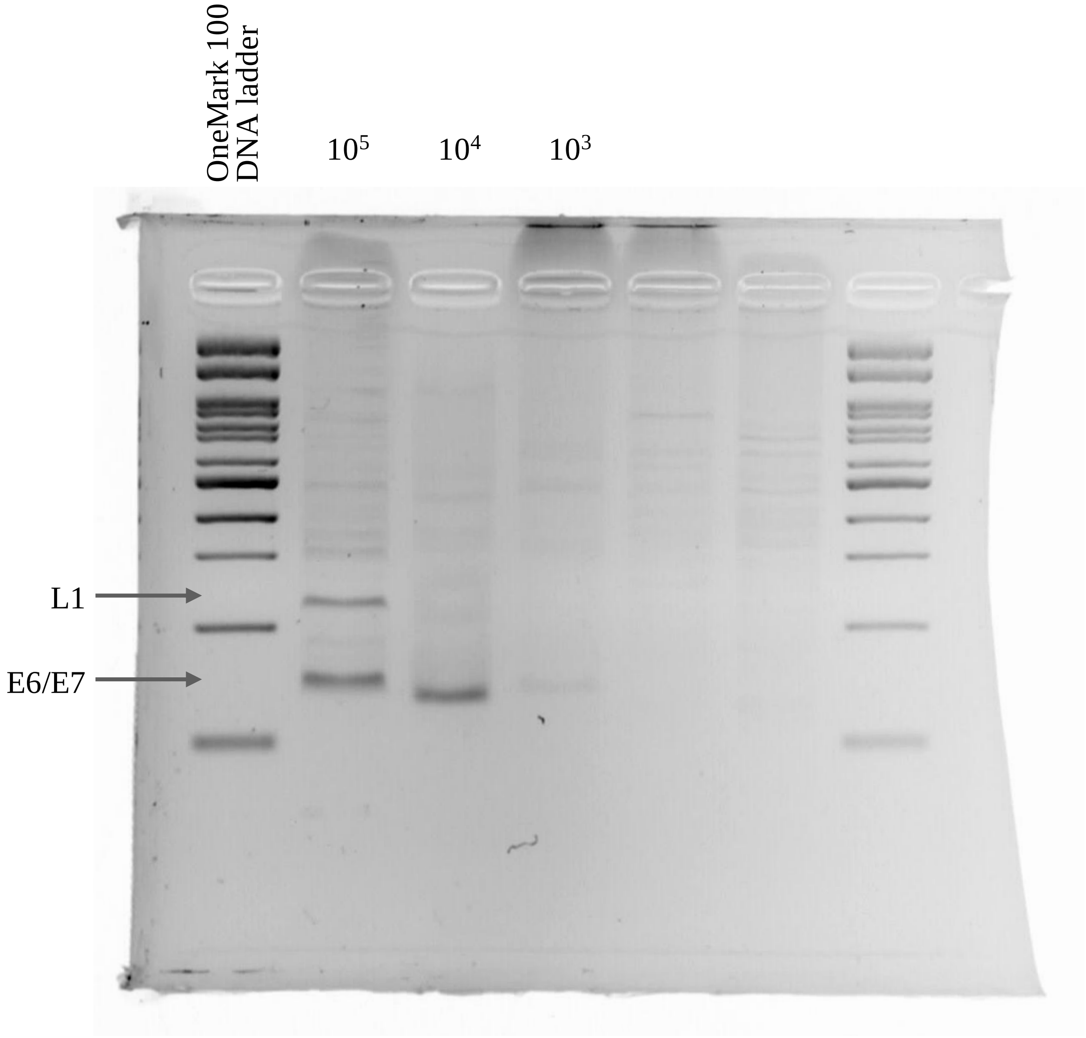

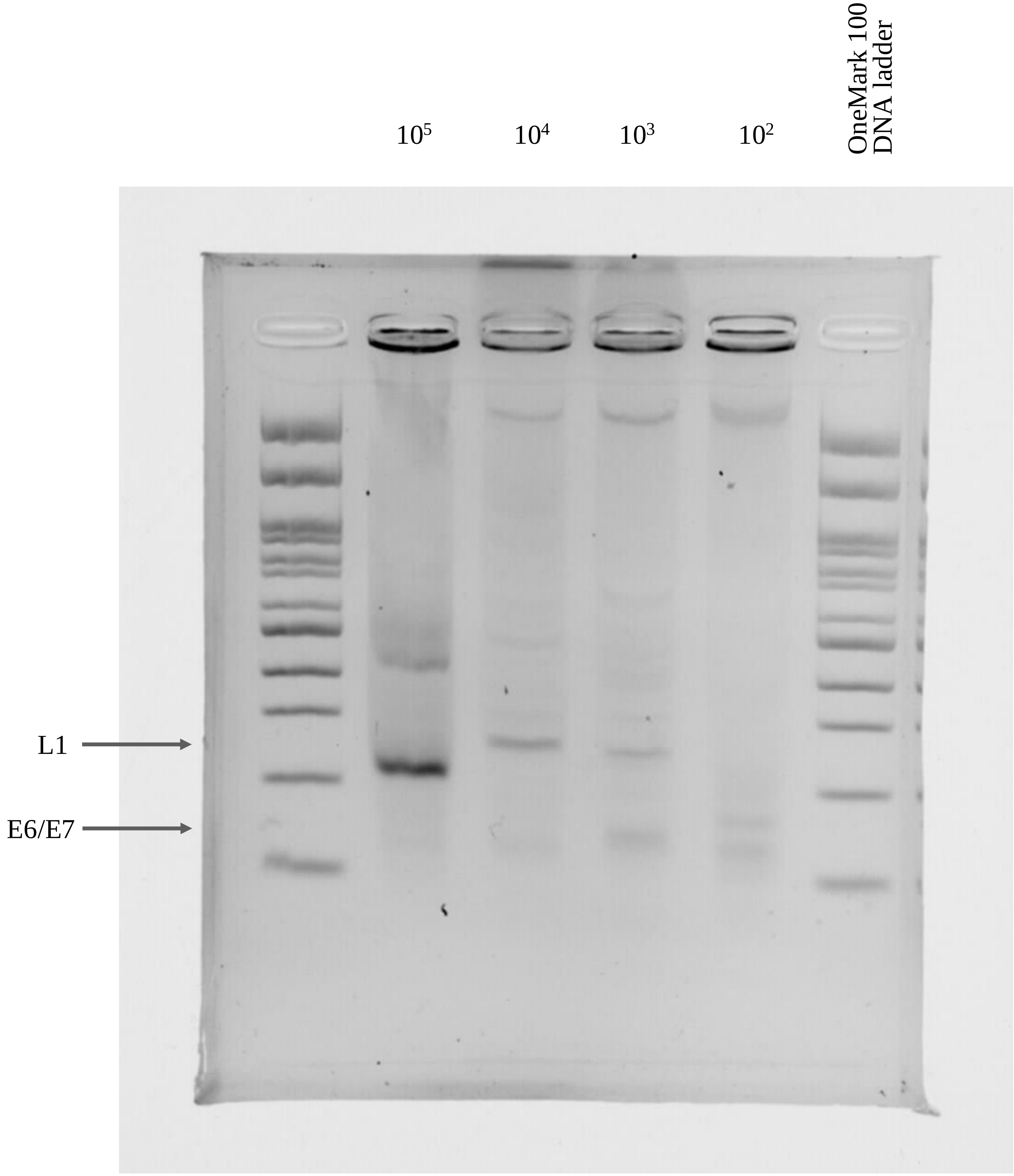
10^5
10^4
10^3
10^2
OneMark 100 DNA ladder
L1
E6/E7
