# Supplementary material for: Multiplex recombinase polymerase amplification for high-risk and low-risk type HPV detection, as potential local use in single tube
Source: Sci Rep. 2023 Jan 16;13:829. doi: 10.1038/s41598-023-28038-9 (PMC9841928; doi:10.1038/s41598-023-28038-9)
Supplement: Supplementary file 3 — Supplementary Information 3. [file 41598_2023_28038_MOESM3_ESM.pdf]

Multiplex recombinase polymerase amplification for high-risk and low-risk type HPV detection, as potential local use in single tube

Rungdawan Wongsamart[a,b], Parvapan Bhattarakasol[c,d], Arkom Chaiwongkot[c,d], Doonyapong Wongsawaeng[e], Pilailuk Akkapaiboon Okada[f], Tanapat Palaga[a], Asada Leelahavanichkul[c], Weerapan Khovidhunkit[g], Deborah Dean[h,i,j], Naraporn Somboonna[a,b,k*]

Supplementary Figures and Tables

Supplemental Figure 1. Minimal multiplex primer concentration for L1 (**A**), E6/E7 (**B**), and both L1 and E6/E7 (**C**) PCR.

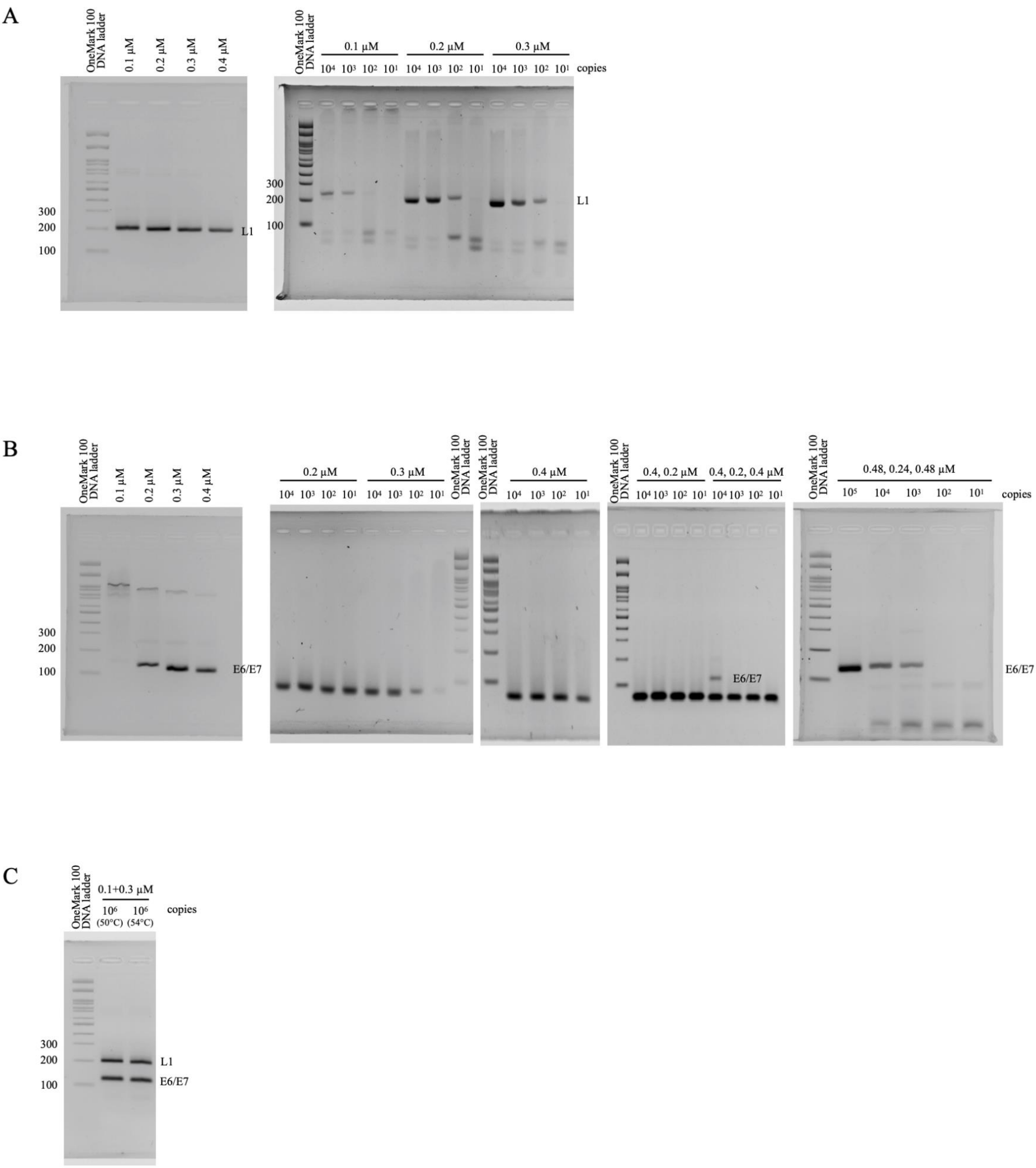
A
OneMark 100 DNA ladder
0.1 µM
0.2 µM
0.3 µM
0.4 µM
300
200
100
L1
0.1 µM
0.2 µM
0.3 µM
10⁴ 10³ 10² 10¹ 10⁴ 10³ 10² 10¹ 10⁴ 10³ 10² 10¹ copies
B
0.2 µM
0.3 µM
0.4 µM
0.4, 0.2 µM
0.4, 0.2, 0.4 µM
0.48, 0.24, 0.48 µM
10⁵ 10⁴ 10³ 10² 10¹ copies
E6/E7
C
0.1+0.3 µM
10⁶ (50°C)
10⁶ (54°C)
copies

Supplemental Figure 2. Agarose electrophoretic images of mRPA reaction at optimal reaction conditions using single E6/E7 template.

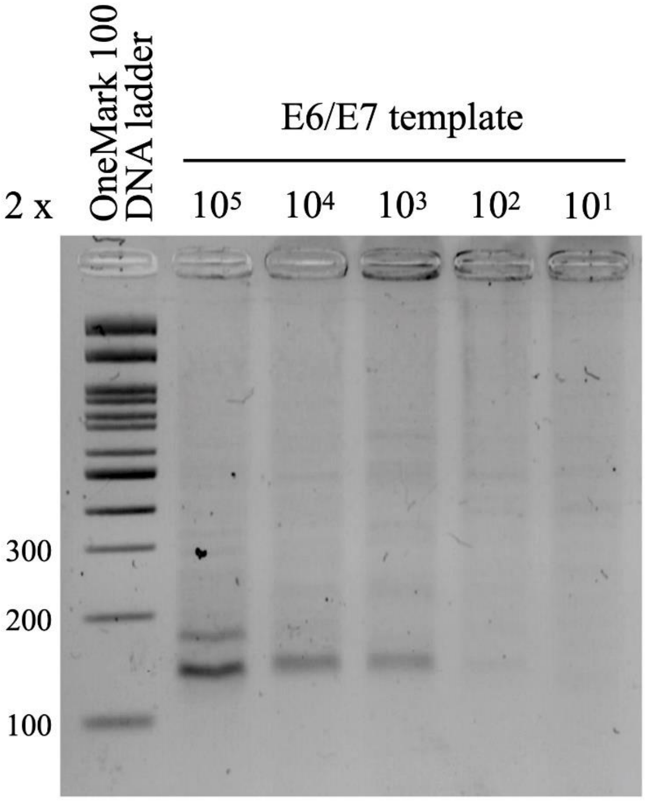

Supplemental Table 1. PCR primers for HPV L1 and E6/E7 genes of HR (types 18 and 33) and LR (11).

Target gene and HPV type	Primer name	Sequences (5'→ 3')	Annealing temp. (°C)	Product size (bp)
L1 HPV18	PCR_ L1-HPV18F	CCTGGCAGCTGTGTGTATTC	63	669
	PCR_ L1-HPV18R	TTACTTCCTGGCACGTACAC		
L1 HPV11	PCR_L1-HPV11F	AGTGGTGGGTATGGTGGT	61	1056
	PCR_ L1-HPV11R	TATACCTGTACGAGCAGACG		
E6/7 HPV18	PCR_E6/7HPV18F	ACAAGCTACCTGATCTGTGC	61	729
	PCR_E6/7HPV18R	AGGACAGGGTGTTCAGAA		
E6/7 HPV33	RPA_HPV_HR_F	GAGGTATATGAYTTTGCTTTTCSWGATTTA	65	492
	E6/7HPV33R	CTGTGGCTGGTTGTDCTTGTCCATCTGGC		
